# Multi-Scale Flexible Fitting of Proteins to Cryo-EM Density Maps at Medium Resolution

**DOI:** 10.3389/fmolb.2021.631854

**Published:** 2021-03-19

**Authors:** Marta Kulik, Takaharu Mori, Yuji Sugita

**Affiliations:** ^1^ Theoretical Molecular Science Laboratory, RIKEN Cluster for Pioneering Research, Wako-shi, Japan; ^2^ RIKEN Center for Computational Science, Kobe, Japan; ^3^ RIKEN Center for Biosystems Dynamics Research, Kobe, Japan

**Keywords:** flexible fitting, multi-scale methods, replica exchange, molecular dynamics simulation, cryo-EM density map, all-atom force field, coarse-grained force field, targeted molecular dynamics

## Abstract

Structure determination using cryo-electron microscopy (cryo-EM) medium-resolution density maps is often facilitated by flexible fitting. Avoiding overfitting, adjusting force constants driving the structure to the density map, and emulating complex conformational transitions are major concerns in the fitting. To address them, we develop a new method based on a three-step multi-scale protocol. First, flexible fitting molecular dynamics (MD) simulations with coarse-grained structure-based force field and replica-exchange scheme between different force constants replicas are performed. Second, fitted Cα atom positions guide the all-atom structure in targeted MD. Finally, the all-atom flexible fitting refinement in implicit solvent adjusts the positions of the side chains in the density map. Final models obtained via the multi-scale protocol are significantly better resolved and more reliable in comparison with long all-atom flexible fitting simulations. The protocol is useful for multi-domain systems with intricate structural transitions as it preserves the secondary structure of single domains.

## Introduction

The technical advances in single-particle cryo-EM result in a rapid escalation of the number of available density maps at increasingly higher resolutions recently crossing the 1.3 Å barrier (Yip et al., 2020) (Nakane et al., 2020). Also, the lower molecular weight limit of this method already reached much below 50 kDa (Wu and Lander, 2020). In order to extract structural information from the experimental densities, computational techniques such as rigid-docking (Wriggers et al., 1999) (Roseman, 2000), flexible fitting (Tama et al., 2004) (Topf et al., 2008) (Trabuco et al., 2008) (Whitford et al., 2011) (Dou et al., 2017) (Mori et al., 2019), and *de novo* modeling (Lindert et al., 2012) (Wang et al., 2015) (Terashi and Kihara, 2018) are essential to assign or refine the atomic positions into those maps. The choice of the computational method depends on the map resolution (Villa and Lasker, 2014). At near-atomic resolution (typically 3-5 Å), the structural details visible in the density give enough confidence for building the models *de novo*, even though the completeness and accuracy of such models would be lower than those based on atomic resolution density maps (Wang et al., 2015) (Zivanov et al., 2018). However, the density maps do not always reach so high resolutions due to intrinsic flexibility or fluctuation of biomolecules, and the methods other than *de novo* building are often required in such cases.

Flexible fitting has been widely used to guide the initial structure toward the target density map, in which normal mode analysis (Tama et al., 2004) or MD algorithms are utilized (Trabuco et al., 2008) (Orzechowski and Tama, 2008). Here, a biasing potential is added to the molecular mechanics force field, and its force constant regulates the magnitude of the fitting. The flexible fitting usually requires a starting structure in a different state or closely related to the one prepared from other methods such as homology modeling. The results of the flexible fitting are strongly system-dependent, often fail for complex conformational changes or for globular-shaped molecules when low- or medium-resolution maps (>5 Å) are treated. This is mainly due to degeneracy of the experimental data, which prevents us from determining a unique conformation (Goh et al., 2016). Another common problem is overfitting, which leads to excessive deformations of the fitted structure and usually occurs when the applied force is too strong. On the other hand, a too weak force constant brings poor results of the flexible fitting. This issue underlines the importance of the optimization of the force constant.

REUSfit is one of the useful methods to overcome this problem (Miyashita et al., 2017), which is an extension of the replica-exchange umbrella-sampling method (REUS) (Sugita et al., 2000). Here, copies of the original system are prepared, and different force constants in the biasing potential are assigned to each replica. The flexible fitting is carried out in parallel, where the force constants are exchanged during the simulation. REUSfit showed better results than simple MD-based flexible fitting, as it enables the automatic adjustment of the force constant (Miyashita et al., 2017). However, REUSfit is computationally expensive if it is employed with the all-atom (AA) model with explicit solvent.

To date, various molecular models at different resolutions have been developed in efficient and accurate MD simulations of proteins, ranging from Coarse-grained (CG) Cα models (Kolinski, 2004) (Kmiecik et al., 2016), through AA models in implicit solvent (Schaefer et al., 1998) (Grochowski and Trylska, 2008) (Onufriev and Case, 2019) and in explicit solvent (Zhou, 2003). CG models are computationally efficient, among which the Go model has been widely used for tackling large conformational changes of proteins (Taketomi et al., 1975) (Whitford et al., 2010) (Kobayashi et al., 2015). Implicit solvent model like the GB/SA model treats solvent molecules as the continuum to reduce the computational cost for the solute-solvent or solvent-solvent interaction calculations (Anandakrishnan et al., 2015). CG model is the most suitable for REUSfit, while high-resolution molecular model is eventually desired to refine the protein structure, as it reveals the atomic interactions in biomolecules at a greater level of detail and accuracy.

In this study, we propose a multi-scale protocol in the cryo-EM flexible fitting that can connect CG and AA models in order to reach the final structural models with a quality not accessible by using any of those simulation methods separately. We focus on medium resolution density maps, i.e., around 5-8 Å, since the flexible fitting is generally useful for such maps. We tested our protocol in various systems and found that it is particularly useful for the protein complexes with large conformational transitions, such as Ca^2+^-ATPase and DNA polymerase.

## Methods

### Flexible Fitting Algorithm

In MD simulations with flexible fitting, biomolecular structure is guided toward the EM density map by a biasing potential *V*_*EM*_. This potential is simply added to the force field function and depends on a given force constant *k* and a cross-correlation coefficient *CC* between the target density map and the one calculated in the course of the simulation (Orzechowski and Tama, 2008):

$$V_{\mathrm{EM}}=k\left(1-\mathrm{CC}\right).$$

CC is computed for each (*i,j,k*) voxel containing the target *ρ* ^*targ*^ and calculated *ρ* ^*calc*^ densities (Tama et al., 2004):

$$\mathrm{CC}=\frac{\sum_{\mathrm{ijk}}\rho^{t\arg}\left(\mathrm{i,j,k}\right)\rho^{\mathrm{calc}}\left(\mathrm{i,j,k}\right)}{\sqrt{\sum_{\mathrm{ijk}}\rho^{t\arg}{\left(\mathrm{i,j,k}\right)}^{2}\sum_{\mathrm{ijk}}\rho^{\mathrm{calc}}{\left(\mathrm{i,j,k}\right)}^{2}}}$$

Introduction of REUS scheme (Sugita et al., 2000) to the flexible fitting simulations enables to dynamically adjust the force constant during the simulation. This approach attributes higher force constants to the replicas with higher *CC*, i.e., better fitted to the target density maps. The exchange probability between two replicas *i* and *j*, with force constants *k*_*m*_ and *k*_*n*_ and CC to the target map equal to *CC* ^*i*^ and *CC* ^*j*^, respectively, is estimated in the following way (Miyashita et al., 2017):

$$w\left(X\to X^{'}\right)\equiv w\left(x_{m}^{\left[i\right]}|x_{n}^{\left[j\right]}\right)=\{\begin{array}{c} 1,\;\;\;\;\;\;\;\;\mathrm{for}\;\Delta \;\leq 0\;\\ \exp\left(-\Delta \right),\;\mathrm{for}\;\Delta \;>0 \end{array}$$

$$\Delta =\beta \left(k_{n}-k_{m}\right)\left(CC^{j}-CC^{i}\right)$$

The parameter β stands for the inverse temperature and here is the same for all replicas. This method was implemented in GENESIS as REUSfit (Miyashita et al., 2017). For details about the generation of density maps with a Gaussian mixture model and about the domain decomposition and atomic decomposition parallelization strategies developed to speed up the flexible fitting simulations, see (Mori et al., 2019).

### Targeted MD Algorithm

In targeted MD, the aim is to simulate the conformational transition of a molecule at initial configuration vector ***r***_*I*_ to the known target state given by configuration vector ***r***_*F*_. For each configuration, a distance between the current and target configuration can be calculated as (Schlitter et al., 1994):

$$\rho =\left|r-r_{F}\right|$$

In order to force the system to undergo the desired transition, we introduce a force constraint *F* ^*c*^:

$$\phi \left(r\right)={\left|r-r_{F}\right|}^{2}-\rho^{2}=0$$

$$F^{c}\equiv \lambda \frac{\mathrm{d\phi}}{\mathrm{dr}}=2\lambda \left(r-r_{F}\right)$$

The Lagrangian multiplier λ is calculated to satisfy the above equations. At every time step, ρ is diminished by ∆ρ until the final value ρ_f_ is reached:

$$\Delta \rho =\frac{\Delta t\left(\rho_{0}-\rho_{f}\right)}{T},$$

where T stands for the total simulation time. In GENESIS, the RMSD is used instead of distance. Therefore, the implemented method is based on internal coordinates rather than on Cartesian coordinates.

### Chosen Systems

Seven different systems, containing PDB structures in at least two different conformations, were selected for flexible fitting simulations using simulated density maps: Ca^2+^-ATPase (Toyoshima and Nomura, 2002) (Toyoshima et al., 2000) (Toyoshima and Mizutani, 2004) (Toyoshima et al., 2007), Na^+^ K^+^-ATPase (Morth et al., 2007) (Kanai et al., 2013), Adenylate Kinase (Müller et al., 1996) (Müller and Schulz, 1992), Ribose Binding Protein (Björkman et al., 1994) (Björkman and Mowbray, 1998), Maltodextrin Binding Protein (Sharff et al., 1992) (Quiocho et al., 1997), Diphtheria Toxin (Bennett et al., 1994) (Bennett and Eisenberg, 1994) and CO Dehydrogenase (Darnault et al., 2003). In the case of simulations with the experimental density maps, two systems were chosen, for which PDB structures in two states, as well as an experimental Cryo-EM density map for one of those states, are available: Magnesium Transporter CorA (Matthies et al., 2016) and DNA Polymerase (Fernandez-Leiro et al., 2015).

### CG Simulations

The MMTSB Go model Web Server was used to prepare the Cα model parameters based on the potential energy function developed by Karanicolas and Brooks (Karanicolas and Brooks, 2003). For the structures containing more than one protein chain, in-home scripts were prepared to combine parameters for different chains, taking into account the inter- and intra-chain interactions. Additionally, the native contact values between τ500 and the tail of PolIIIα were increased threefold to prevent τ500 from detaching from the polymerase complex, as this interaction was previously reported stable upon the studied conformational transition (Fernandez-Leiro et al., 2015). The initial and target PDB structures were superimposed using the Cα atoms. The simulated density maps were generated at 5 Å resolution with 2 Å voxel size using the target PDBs and emmap_generator tool from the GENESIS package (Mori et al., 2019). The resolution and voxel size of the experimental density maps were left unchanged and the negative values were removed with the SITUS voledit tool (Wriggers et al., 1999).

The flexible fitting molecular dynamics simulations with replica exchange (REUSfit) were performed using 32 replicas at force constants 500; 524; 555; 593; 636; 684; 737; 794; 856; 921; 991; 1,065; 1,144; 1,228; 1,318; 1,415; 1,519; 1,632; 1,755; 1,889; 2,035; 2,195; 2,371; 2,564; 2,777; 3,011; 3,269; 3,553; 3,865; 4,209; 4,586; 5,000 kcal/mol. The distribution of the force constants was based on a preliminary survey to ensure the sufficient overlap of the biasing potential between replicas. The cutoff distance for the native and non-native contact interaction terms was set to 20 Å. All bond lengths were fixed with the SHAKE algorithm (Ryckaert et al., 1977). The temperature was kept constant at 200 K using the Berendsen thermostat (Berendsen et al., 1984). The exchanges were attempted every 2 ps and each replica was simulated for 250 ns with a time step of 20 fs, except for DNA Polymerase, where 10 fs was used as a time step. The density map was recalculated at every time step with the truncation of the Gaussian function to zero for the densities below 1% of the maximum value (Tama et al., 2004). The REUSfit of each system were repeated 20 times with different initial velocities. The highest CC structures in each simulation were chosen as the best models in each run.

### AA Simulations

CHARMM C36 force field parameters for proteins (Best et al., 2012) were prepared using psfgen plugin in VMD (Humphrey et al., 1996) with the patches for disulfide bonds where necessary. GBSA implicit solvent model with OBC2 parameters (Onufriev et al., 2004) was used with salt concentration 0.15 M and the surface tension coefficient 0.005 kcal·mol^−1^·Å^−2^. The dielectric constant was set to 78.5. For the AA simulations, the 1 Å voxel size of the simulated density maps was used. The experimental density maps were the same as at CG level. In the second step of the multi-scale protocol, the Cα positions from 5 best models from CG simulations were used as a target in targeted MD simulations of AA structures in implicit solvent. The simulations in NVT ensemble with Langevin thermostat with the friction coefficient gamma set at 5 ps^−1^ were performed for 1 ns with time step 2 fs. The final structure after targeted MD will have low RMSD value with respect to the target but we perform additional alignment using the Cα atoms to shift the center of mass of the system.

The last step of the multi-scale simulations is the refinement of the last frame of the targeted MD trajectories with the flexible fitting simulations to the density maps. The simulation is performed for 1 ns using a similar set of parameters as the Targeted MD simulation. For comparison, the AA flexible fitting simulations starting from original PDB structure are carried out in 5 replicates and referred to as the simple protocol. The best models from each of those simulations are chosen according to the highest CC. All simulations were done in GENESIS version 1.4 (https://www.r-ccs.riken.jp/labs/cbrt/).

### Analysis

Various tools were used for the analysis of the best final models M1, M2, M3, SP and also S0 and RF: CC without rigid body docking was calculated in the collage program from Situs package (Wriggers et al., 1999); RMSD in Gromacs 2016.4 (Abraham et al., 2015) with gmx rms tool; CaBLAM and Ramachandran outliers and MolProbity score in Phenix (Williams et al., 2018) (Adams et al., 2010).

CaBLAM and Ramachandran outliers and MolProbity score depend on the relation between the geometry of the final models and a high-quality protein dataset from PDB database, called Top8000. CaBLAM is a method to evaluate the geometry of the protein backbone using the virtual angles and dihedrals created by the Cα atoms and carbonyl groups [details in (Williams et al., 2018)]. Ramachandran outliers use the φ and ψ angles of the protein backbone for analysis.

Secondary structure score is determined by comparing the secondary structure of the fitted model and the target, namely by counting the Hamming distance between two strings, representing the coded secondary structure features for each residue, as calculated by mkdssp version 2.2.1 (Touw et al., 2015) (Kabsch and Sander, 1983) and in-house scripts. The Hamming distance is normalized by dividing it by the length of the string and representing it as a percentage.

## Results

### Multi-Scale Protocol for Flexible Fitting

**FIGURE 1:**
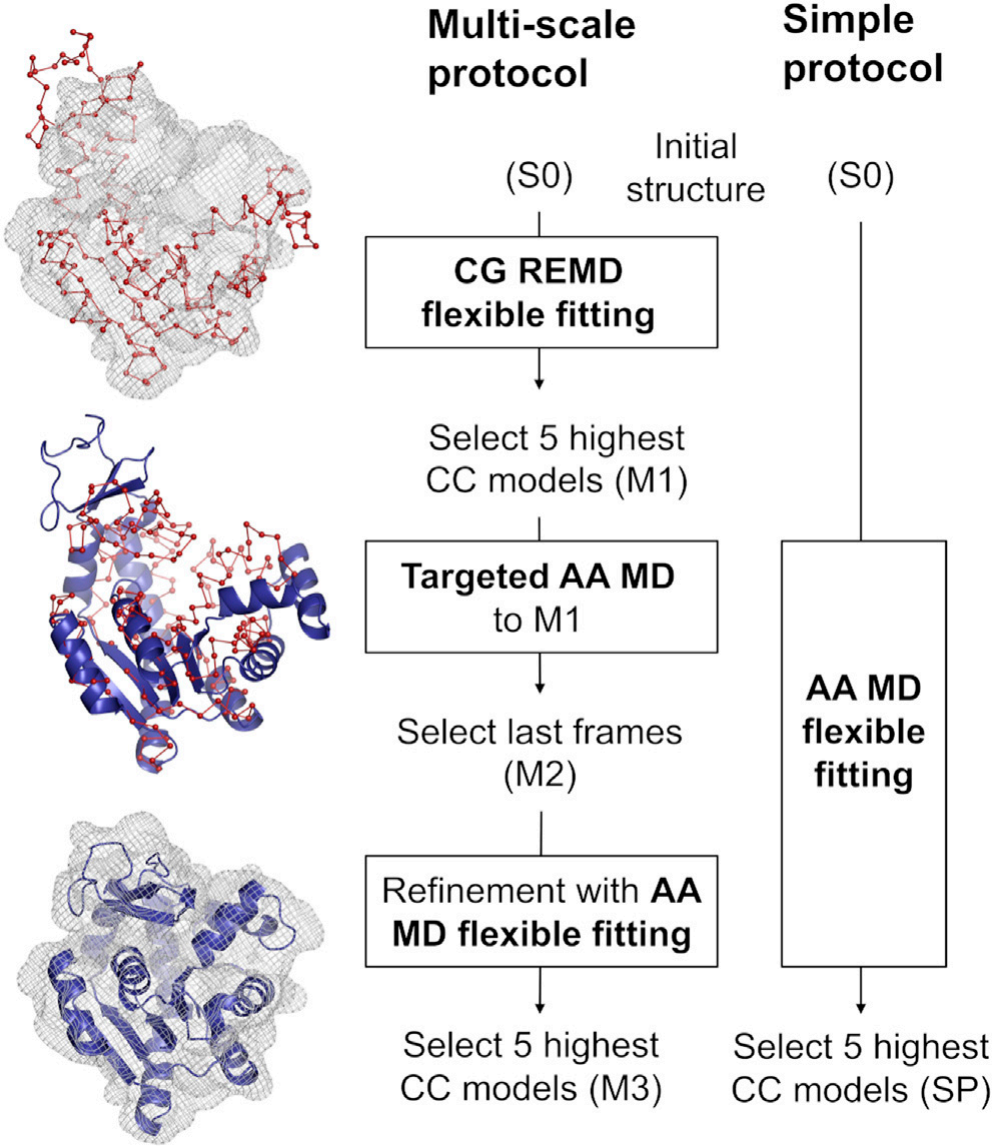
Comparison between the multi-scale protocol and a simple flexible fitting protocol. The positions of Cα atoms in the CG model are shown as red spheres joined by a ribbon representing the pseudobonds. The blue cartoon depicts the protein modeled with AA in implicit solvent. Density map is shown in wireframe representation.

Our multi-scale protocol involves three steps (Figure 1, **left scheme**). In step 1, REUSfit with the Cα Go model (Karanicolas and Brooks, 2003) is employed. In this model, one amino acid is represented by a single bead at their Cα atom position, and the beads interact via the structure-based potential that is constructed from the native structure. The interactions between side chains are scaled for respective amino acid types using Miyazawa-Jernigan contact energies (Miyazawa and Jernigan, 1996). In REUSfit, the cross-correlation-coefficient (CC) between the calculated and experimental density maps is included in the biasing potential. We carry out five individuals runs, and the best models, called M1, are chosen from each run according to the highest CC. In step 2, we carry out targeted MD (Schlitter et al., 1994) starting from the initial atomic structure toward M1, where the implicit solvent model (GB/SA) (Onufriev et al., 2004) is used to reduce the computational cost. Note that the experimental density map is not used in this step. Since targeted MD imposes the restraint on the root-mean-square deviation (RMSD) with respect to the target structure, the Cα coordinates in the AA model do not exactly match the target CG model. Therefore, additional alignment to M1 is necessary to shift the center of mass of the obtained structure after the targeted MD. The final atomic structure is called M2. In step 3, M2 is further refined with the CC-based flexible fitting using the experimental density map. Here, the GB/SA implicit solvent model is again used as in step 2. The final models are chosen according to their CC and named M3.

We compare the best final models obtained via two protocols, starting from the same initial structure S0: the multi-scale protocol and one, long, all-atom flexible fitting simulation called a simple protocol (SP) (Figure 1, **right scheme**). The computational condition is same as in step 3 of the multi-scale protocol.

The two protocols are applied to a range of systems with simulated and experimental density maps, shown in Table 1. Simulated density maps serve as a test of accuracy and precision of fitting. The density maps were generated from X-ray crystal structures of the target biomolecules at 5 Å resolution. Tested systems include a small protein such as Adenylate Kinase as well as relatively large ones, for instance, DNA polymerase. All tested systems are classified as having two significantly different states in initial and target structures. The transitions between the two states represent varying levels of difficulty in each case. Particularly, Ca^2+^-ATPase and Diphtheria Toxin are difficult to fit correctly (initial RMSD >10 Å), while Ribose Binding Protein and Maltodextrin Binding Protein are relatively easy (initial RMSD <4 Å). For the experimental density maps, we have chosen two systems, for which both the cryo-EM density maps and fitted models were available, together with a starting structure in a different physiological state, involving complex conformational transition between the two states. Two systems fulfilling all the criteria were found, with cryo-EM density maps at resolution in the range 5-8 Å: magnesium transporter CorA (Matthies et al., 2016) and DNA polymerase (Fernandez-Leiro et al., 2015).

**TABLE 1:** Systems used in the flexible fitting simulations using either simulated or experimental density maps. The RMSD was calculated between the Cα atoms of the initial structure and the target structure, for the number of residues (and chains), specified in the last column.

System	State (PDB ID) of the initial → target structures	Density map, resolution [Å]	RMSDt (Cα) [Å]	# of residues (chains)
Ca^2+^-ATPase	E2 (1iwo) → E1∙2Ca^2+^ (1su4)	simulated, 5.0	14.0	994 (1)
	E1∙2Ca^2+^ (1su4) → E1∙ATP (1vfp)	simulated, 5.0	13.7	994 (1)
	E1∙ATP (1vfp) → E2P (2zbf)	simulated, 5.0	11.2	994 (1)
	E2P (2zbf) → E2 (1iwo)	simulated, 5.0	5.5	994 (1)
Na^+^ K^+^-ATPase	K^+^∙E2 (3kdp) → Na^+^∙E1P∙ADP (3wgu)	simulated, 5.0	11.6	1,280 (2)
	Na^+^∙E1P∙ADP (3wgu) → K^+^∙E2 (3kdp)	simulated, 5.0	11.6	1,280 (2)
Adenylate kinase	open (4ake) → closed (1ake)	simulated, 5.0	7.1	214 (1)
Ribose binding protein	closed (2dri) → open (1urp)	simulated, 5.0	4.1	271 (1)
Maltodextrin binding protein	open (1omp) → closed (1anf)	simulated, 5.0	3.8	370 (1)
Diphtheria toxin	open (1ddt) → closed (1mdt)	simulated, 5.0	15.6	523 (2)
CO dehydrogenase	closed (1oaoC) → open (1oaoD)	simulated, 5.0	7.9	729 (1)
Magnesium transporter CorA	closed (3jcf) → open (3jcg)	Experimental (EMD-6552), 7.1	9.7	1,653 (5)
DNA polymerase	Bound (5fkv) → free (5fku)	Experimental (EMD-3201), 8.3	14.2	2,211 (7)

### Large Conformational Transitions can be Fitted Well With Multi-Scale Protocol

First, we focus on Ca^2+^-ATPase, which requires large conformational transitions during the flexible fitting. Ca^2+^-ATPase undergoes a series of structural changes between E2, E1∙2Ca^2+^, E1P, and E2P states (Supplementary Figure S1), linked with Ca^2+^ transport against the concentration gradient (Toyoshima, 2008). If the flexible fitting is performed in these states, the movements of cytoplasmatic domains (A, P, and N) and transmembrane helices in domain M are challenging for us, since the A domain rotates by 90° from the E1P to E2P state and the inclination of the N domain with respect to the P domain in the E1·2Ca^2+^ and E1·AMPPCP crystal structures changes by almost 90° as well.

**FIGURE 2:**
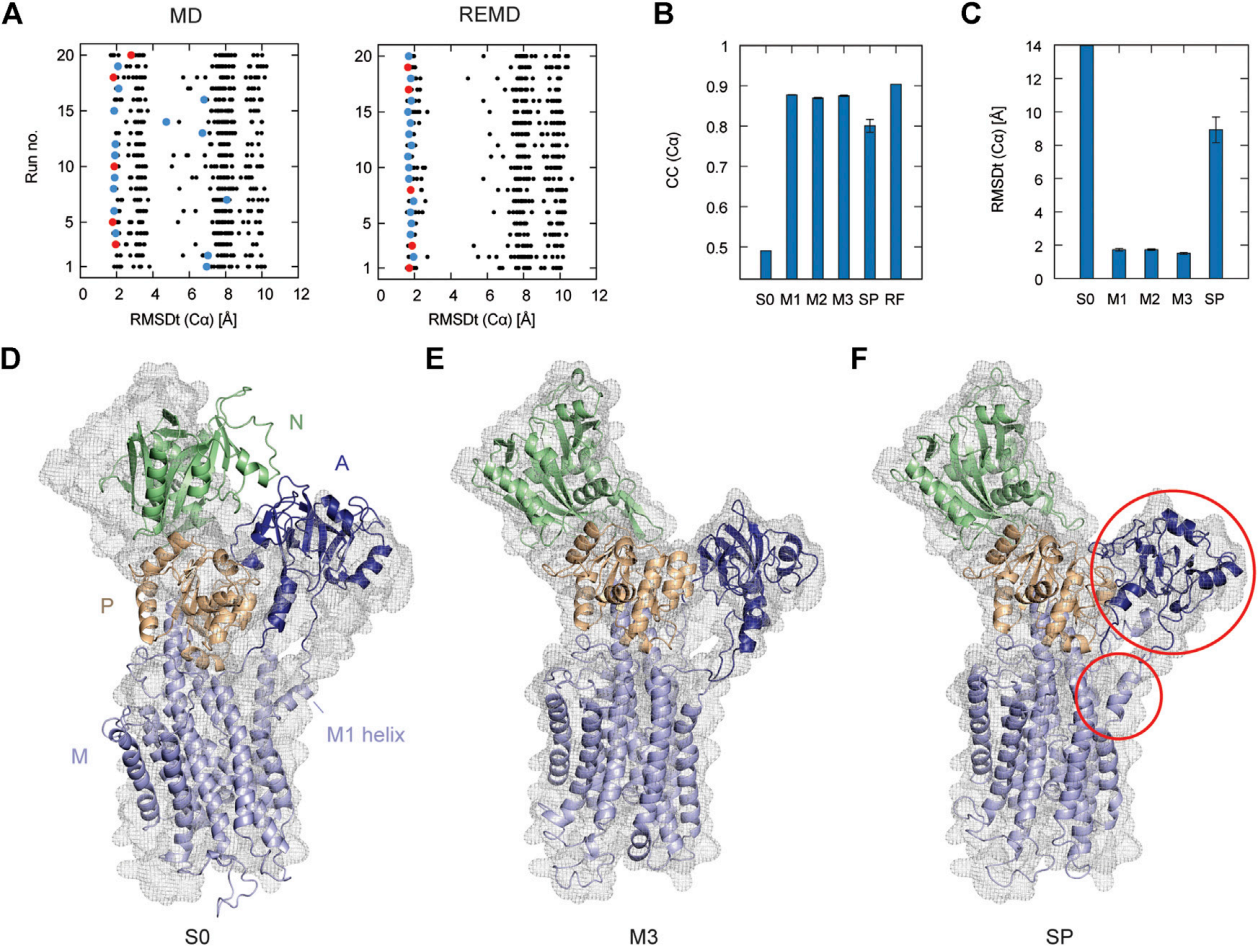
Flexible fitting of Ca^2+^-ATPase from E2 to E1∙2Ca^2+^ state. **(A)** RMSDt of Cα atoms of best CC models of each of 32 MD replicas at different force constants for 20 independent simulation runs. The best CC replica in each run is colored and the chosen 5 best models are highlighted in red. MD and REMD panels show simulations without and with exchanges between replicas, respectively. **(B)** Average CC of the initial structure (S0) and 5 best models in the multi-scale protocol stages: CG REMD simulations (M1), targeted MD (M2), AA MD (M3) and in the simple protocol (SP). Standard deviation is shown as an error bar. **(C)** RMSDt of the (Cα) atoms of the same structures. **(D)** Initial structure used for simulations, superimposed on the target density map. **(E**, **F)** The highest CC model obtained using the multi-scale protocol and simple protocol, respectively.

In Figure 2A, the left panel shows 20 individual runs of the flexible fitting from E2 to E1∙2Ca^2+^ states using the CG model at different force constants (500–5,000 kcal/mol), which corresponds to REUSfit without replica exchange. The final fitted models with the highest CC are colored and chosen as the best models. Their RMSD with respect to the target structure (RMSDt) around 2 Å represent the successful simulations with well-fitted domain A. However, there are multiple failed attempts. Introducing the exchanges between the replicas brings substantial improvement, and all the attempts are successful (right panel in Figure 2A). Each fitting run of the multi-scale protocol is highly repeatable due to exchanges between replicas. Therefore, a single fitting simulation following the multi-scale procedure is sufficient to obtain a reliable result, which is visible in the low values of standard deviation of multiple replicates in Table 2. Best models from the simple protocol reveals significantly higher standard deviations. Similar results were obtained in the previous work using 10 Å resolution maps (Miyashita et al., 2017). Thus, the structures with the correct Cα atoms positions (M1) can be further used in the multi-scale protocol in targeted MD and refined to obtain M2 and M3 models, respectively.

**TABLE 2:** Quality of the best models obtained using the simple and multi-scale protocol for the simulated density maps for Ca^2+^-ATPase and Diphtheria Toxin.

System	Str.	c.c.	RMSDt (Cα) [Å]	RMSDt (AA) [Å]	MolProbity score	Ramachandran outlier [%]	Rotamer [%]	CABLAM [%]	Sec. struct. index [%]
Ca^2+^ ATPase									
E2 → E1∙2Ca^2+^	MS3	0.96 (0.95 ± 0.00)	1.4 (1.5 ± 0.0)	2.3 (2.4 ± 0.1)	1.67 (1.74 ± 0.05)	2.7 (3.3 ± 0.3)	6.3 (7.3 ± 0.7)	4.0 (5.3 ± 1.0)	22 (23 ± 1)
	SP	0.91 (0.90 ± 0.02)	8.8 (8.9 ± 0.8)	9.2 (9.4 ± 0.8)	1.85 (1.80 ± 0.04)	4.2 (4.8 ± 0.4)	8.5 (8.7 ± 0.7)	7.2 (7.5 ± 1.3)	31 (32 ± 1)
E1∙2Ca^2+^ → E1∙ATP	MS3	0.96 (0.95 ± 0.00)	1.5 (1.5 ± 0.1)	2.3 (2.3 ± 0.1)	1.79 (1.82 ± 0.03)	2.3 (2.4 ± 0.4)	9.1 (9.7 ± 1.1)	4.7 (4.0 ± 0.6)	21 (23 ± 1)
	SP	0.94 (0.94 ± 0.00)	3.0 (2.8 ± 0.3)	3.6 (3.5 ± 0.2)	1.83 (1.82 ± 0.05)	3.7 (3.6 ± 0.4)	9.0 (8.8 ± 1.0)	5.1 (5.8 ± 0.5)	22 (24 ± 1)
E1∙ATP → E2P	MS3	0.96 (0.96 ± 0.00)	1.4 (1.4 ± 0.1)	2.3 (2.3 ± 0.1)	1.72 (1.75 ± 0.06)	2.5 (2.7 ± 0.4)	8.5 (7.9 ± 0.7)	2.8 (3.7 ± 0.5)	21 (21 ± 1)
	SP	0.93 (0.92 ± 0.02)	4.4 (5.1 ± 1.5)	4.9 (5.6 ± 1.5)	1.85 (1.84 ± 0.04)	4.3 (4.0 ± 0.7)	9.4 (8.4 ± 0.8)	6.3 (6.5 ± 0.8)	26 (28 ± 1)
E2P → E2	MS3	0.96 (0.96 ± 0.00)	1.3 (1.3 ± 0.0)	2.1 (2.1 ± 0.0)	1.56 (1.69 ± 0.02)	2.0 (2.0 ± 0.3)	6.5 (7.4 ± 0.6)	2.9 (3.2 ± 0.6)	21 (23 ± 1)
	SP	0.96 (0.95 ± 0.00)	1.4 (1.5 ± 0.1)	2.2 (2.3 ± 0.1)	1.63 (1.70 ± 0.05)	1.7 (2.1 ± 0.4)	6.5 (7.7 ± 1.2)	3.6 (3.7 ± 0.6)	24 (25 ± 2)
Diphtheria toxin	MS3	0.97 (0.97 ± 0.00)	0.8 (0.9 ± 0.1)	1.6 (1.7 ± 0.1)	1.71 (1.74 ± 0.03)	2.7 (3.0 ± 0.3)	7.8 (7.7 ± 1.0)	5.4 (5.2 ± 0.5)	18 (19 ± 2)
	SP	0.95 (0.95 ± 0.00)	9.9 (9.6 ± 0.3)	10.3 (9.9 ± 0.3)	1.93 (1.86 ± 0.05)	6.0 (5.4 ± 0.6)	8.3 (7.7 ± 0.5)	10.5 (8.9 ± 1.5)	38 (33 ± 3)

In Figure 2B, we summarize the results of the multi-scaling protocol, comparing the averaged CC of the obtained M1, M2, and M3 models. For comparison, CC of the initial structure S0, reference structure RF, and the results of the simple protocol (SP) are also shown. The best models obtained using our multi-scale protocol (CC = ∼0.88) are significantly better than that in the simple protocol (CC = ∼0.80), indicating a successful fitting to the density map. RMSDt of the M3 model is also smaller (1.5 Å) than that of the SP model (8.9 Å) (Figure 2C). In addition, the MolProbity score (Keedy et al., 2009), CaBLAM (Williams et al., 2018), and Ramachandran outliers of the M3 model are better than the SP model (Supplementary Figure S2). The RMSDt of the SP models are closer to the S0 structure, implying that during the single-step fitting, smaller conformational changes occurred than during the three-step multi-scale protocol. In fact, RMSDi (RMSD with respect to the initial structure) of the M2 and M3 models is larger than that of the SP model (Supplementary Figure S2).

In the multi-scale protocol, all three steps show similar averaged values of the CC (Cα) and RMSDt (Cα), around 0.88 and 1.5–1.7 Å, suggesting that the first CG simulation with REUSfit is the most important to determine the positions of the Cα atoms. Targeted MD is also working well to superimpose the all-atom model to the CC model by means of a gradual decrease of the RMSD of Cα atoms to their target positions. The protein side chains follow the conformational changes induced by the shift of Cα atoms. The detailed conformation of the protein side chains can be refined in step3, as the density map is not present during the targeted MD. We found that RMSDt of all non-hydrogen atoms is improved from 2.7 to 2.4 Å (Supplementary Figure S2). Although each domain undergoes almost rigid-body rotation and thus most side chains are already well positioned in the targeted MD, further refinement seems to be possible in step3.

One of the main differences between the M3 and SP models is the orientation of the A domain. In the initial structure, the N and A domains are contacting each other (Figure 2D), and they are separated during the fitting. The A domain acts as an actuator of the gating of the ion pathway and it has to rotate in order to regulate the Ca^2+^ binding and release. In the CG simulation, this shift occurs with a domain rotation. This success extends to the all-atom level in the next stage of the multi-scale protocol, resulting in a correct final model (Figure 2E and Supplementary Movie S1). On the other hand, in the simple protocol the A domain was simply fitted to the density without correct rotation, and none of the simulations was successful (Figure 2F and Supplementary Movie S2). Since the orientation of the A domain can affect the transmembrane domain through the loop connecting them, the conformation of the N-terminal side of the M1 helix is also reproduced in the M3 model.

Interestingly, troublesome regions in the flexible fitting in the other physiological states of Ca^2+^-ATPase and Na^+^ K^+^-ATPase are similar, including the transmembrane M1 and M2 helices and A domain. (Supplementary Figure S3). In these cases, the multi-scale protocol still performs significantly better than the simple protocol (see Table 2 below). The main reason behind this is the rotation of the A domain between E1 and E2-type states, performed correctly in the multi-scale protocol and incorrectly in the simple protocol. Without the proper rotation, the secondary structure of the A domain in SP models becomes deformed to accommodate to the density map. In the transition from E1∙2Ca^2+^ to E1∙ATP state, the transmembrane helices M1 and M2 undergo shifting and rearrangement with bending of the N-terminal of the M1 helix. This is reasonably well reflected in M3 models but not in SP models, where M1 helix is bent in opposite way than expected.

In order to represent the degree of disruption of the secondary structure of a protein, we are introducing in this work a new measure called a “secondary structure score.” This score is based on a Hamming distance between two strings, containing the coded secondary structure elements of each residue of two structures that are compared (see the details in the Methods section). The smaller is the value of the secondary structure score, normalized and indicated as a percentage, the more similar are two structures. The last panel of the Supplementary Figure S2 shows the secondary structure scores calculated between the target structure and each model M2, M3 and SP. Here, it is well visible that the secondary structure of the best SP model (31%) deviates much more from the target structure than the best M3 model (22%).

**FIGURE 3:**
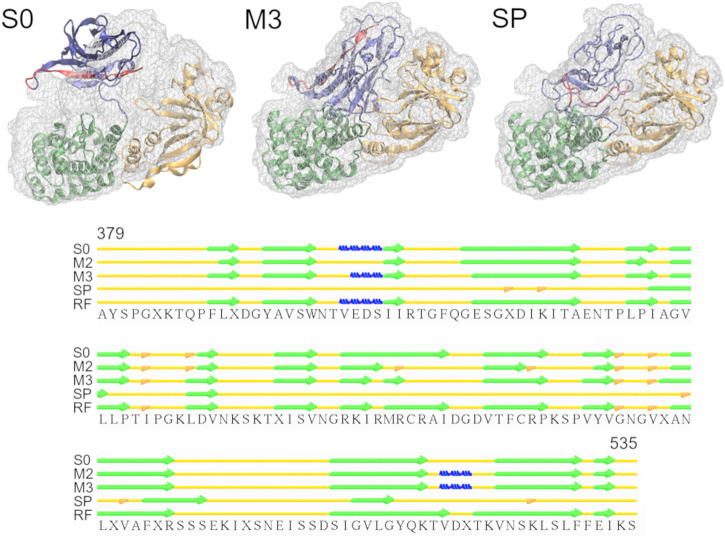
Simulations of Diphtheria Toxin. Initial structure (S0), the highest CC models obtained using the multi-scale protocol (M3) and the simple protocol (SP) are superimposed with the target density map. The R domain is shown in blue with residues 520-535 highlighted in red. The graph below depicts the secondary structure elements in the R domain of the structures from M3, SP and the reference structure (RF), as assigned by the Stride web server. β-strands, 3-10 helices and isolated β-bridges are shown in green, blue and orange, respectively.

Diphtheria Toxin also requires correct domain rotation during the fitting. The initial structure is shown in the S0 panel in Figure 3, where RMSDt is 15.6 Å. To emphasize the change in orientation of the relevant domain (R domain), its C-terminal fragment was highlighted in red. The M3 and SP panels are the highest-CC models obtained from the multi-scale and simple protocols, respectively. The M3 model showed correct orientation of the domain R [average RMSDt (Cα) = 0.9, RMSDi (Cα) = 15.6, and CC (Cα) = 0.89], while it was wrong in the SP model [average RMSDt (Cα) = 9.6, RMSDi (Cα) = 11.7, and CC (Cα) = 0.84]. In addition, there was a disruption of the secondary structure of several β-strands in the R domain of the SP model (bottom panel in Figure 3 and Supplementary Figure S4, average secondary structure score = 33%). Such disruption is not observed in the multi-scale protocol (average sec. struct. score = 19%), presumably because the CG simulations with the Go model enable keeping the native contacts within the domain. A slight improvement of the secondary structure during the final refinement in the multi-scale protocol may be caused by the adjustments of the side chains into the density map, which may influence the backbone geometry and hydrogen bonds between strands.

### Simple Conformational Changes in Small Proteins do not Require Multi-Scale Procedure

The flexible fitting simulations were also performed for several small systems: Adenylate Kinase, Maltodextrin Binding Protein, Ribose Binding Protein, and CO Dehydrogenase. Even though the two conformational states of those systems are classified as significantly different and referred to as “open” and “closed” states in the literatures, the overall transition between them is very simple and does not involve large rotation or shift in the component domains. For those systems, the best models obtained from the multi-scale protocol are similar to those from the simple protocol (Supplementary Figure S5). As a representative, let us take Ribose Binding Protein. The CC (Cα) in the M1, M2, M3, SP, and RF models is 0.89–0.90, and the RMSDt (Cα) is ∼0.6 Å. We found that the multi-scale protocol gives structures of a comparable quality in terms of the MolProbity scores. The average score is 1.34 ± 0.11 in M3, while 1.23 ± 0.08 in SP (see Supplementary Table S1). The average values of Ramachandran, rotamer and CaBLAM outliers are similar within the standard deviation. However, the best M3 models selected basing on the CC are slightly better than the best SP models. Although the multi-scale protocol is applicable to small proteins, the simple protocol seems to be enough in most cases. The multi-scale protocol is useful to search large conformational space. Involving the CG models in the protocol is much more beneficial for large proteins and their complexes.

### The Multi-Scale Protocol Performs Well for Experimental Cryo-EM Density Maps

CorA is responsible for magnesium uptake in prokaryotes. In the closed Mg^2+^-bound state, it forms a symmetrical pentamer. While moving to the open Mg^2+^-free state, the 5-fold symmetry is lost. The density map of the latter state is used here as the target of flexible fitting simulations. In order to assess the performance of our protocol by calculating RMSDt, we have used the model fitted by rigid body methods and deposited by the density map authors in the RCSB PDB database. We realize that this model may not be highly accurate due to its low resolution but that is the only “answer” model that could be used for comparison and we may possibly contribute to its refinement.

**FIGURE 4:**
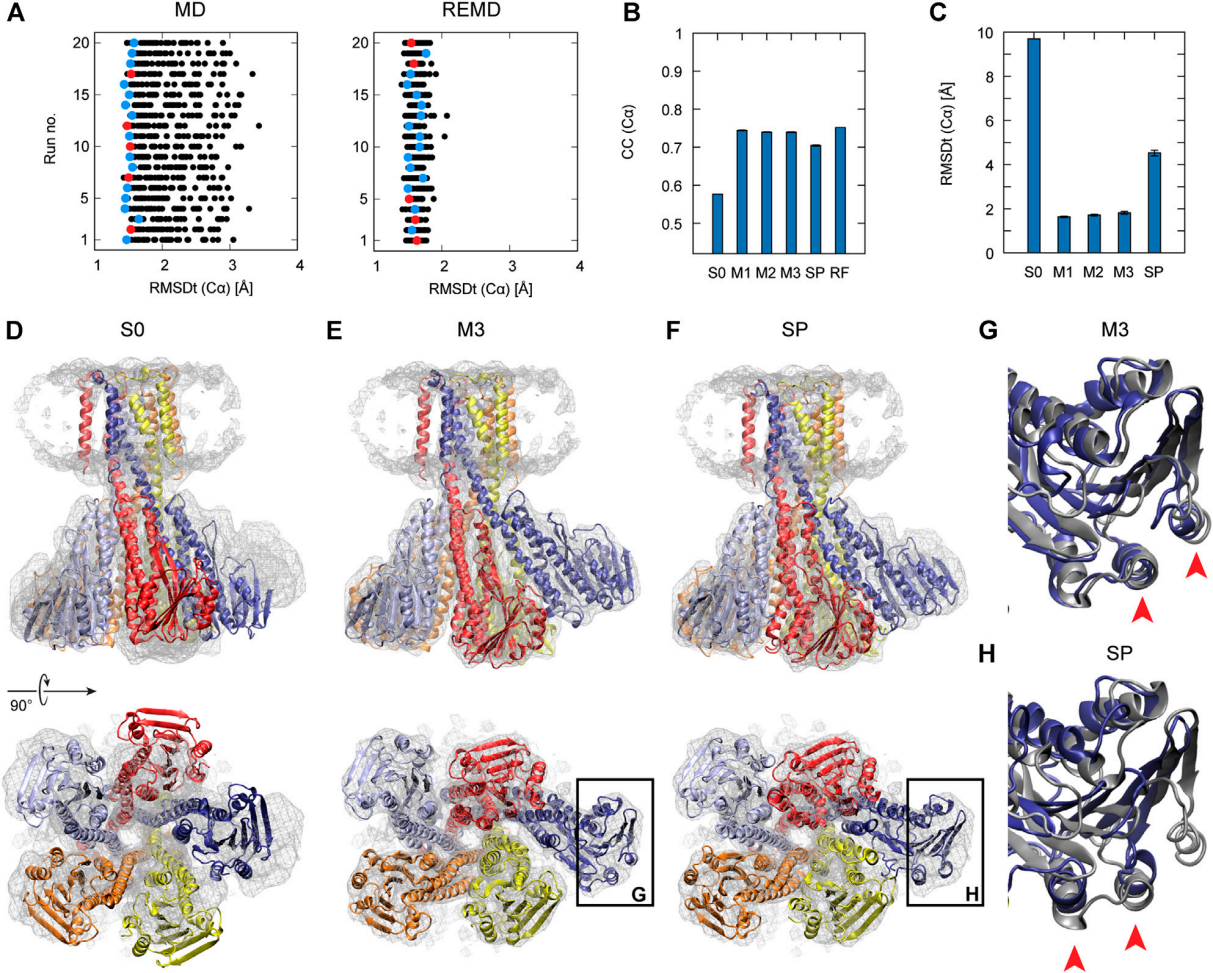
Fitting of Magnesium Transporter CorA to experimental density map. **(A)** RMSDt of Cα atoms of best CC models for runs of simulations without (MD) and with exchanges between replicas (REMD), respectively. 5 best models are highlighted in red. **(B)** Average CC of the initial structure (S0) and 5 best models in the multi-scale protocol stages (M1, M2, M3) and in the simple protocol (SP) with standard deviation as an error bar. **(C)** Average RMSDt of the (Cα) atoms. **(D)** The side view and bottom view of the initial structure with the target density map. **(E)** The highest CC models obtained using the multi-scale and **(F)** simple protocol. Panels **(G**, **H)** show the enlarged view on the chain A with the reference structure in dark gray.

To examine the usefulness of REUSfit in the multi-scale protocol, we first compare CG MD with and without replica exchange. In Figure 4A, we see that REUSfit produced all the models in the RMSDt range 1–2 Å, suggesting that we can select a reliable model even from one simulation without considering the effect of the force constant. Since the targeted MD worked well in step 2, the all-atom model was superimposed to the CG model effectively. As a result, the multi-scale protocol gives significantly better models than simple protocol in terms of both CC (Cα): 0.74 vs 0.70 and RMSDt (Cα): 1.8 vs 4.5 Å (Figures 4B,C). Similar tendency was obtained in the averaged RMSDt for non-hydrogen atoms, CaBLAM outliers, MolProbity score, and secondary structure score (Figure S7). The reason behind it is the difficulty of fitting is the skew of chain A (dark blue in Figures 4D–F). The comparison of two chosen helices of chain A with the target structures shows that they were fitted correctly by the multi-scale protocol in the CG step of the protocol using REUSfit and trapped in a wrong density patch in the best model obtained with the simple protocol (red arrows in Figures 4G,H). The REUSfit method is able to rescue the system from being trapped in local density minima by the efficient conformational sampling.

Step 3 did not improve the structure with respect to the target model, as both CC and RMSDt values of the M2 and M3 models are similar. As already mentioned, the target model is not a high-resolution structure of the system. Also, the resolution of the density map is 7.1 Å. Therefore, it is difficult to conclude whether the final refinement step brings any improvement to the structure. It is expected that for the target density maps of higher resolution, step 3 would contribute more to the quality of the structure.

**FIGURE 5:**
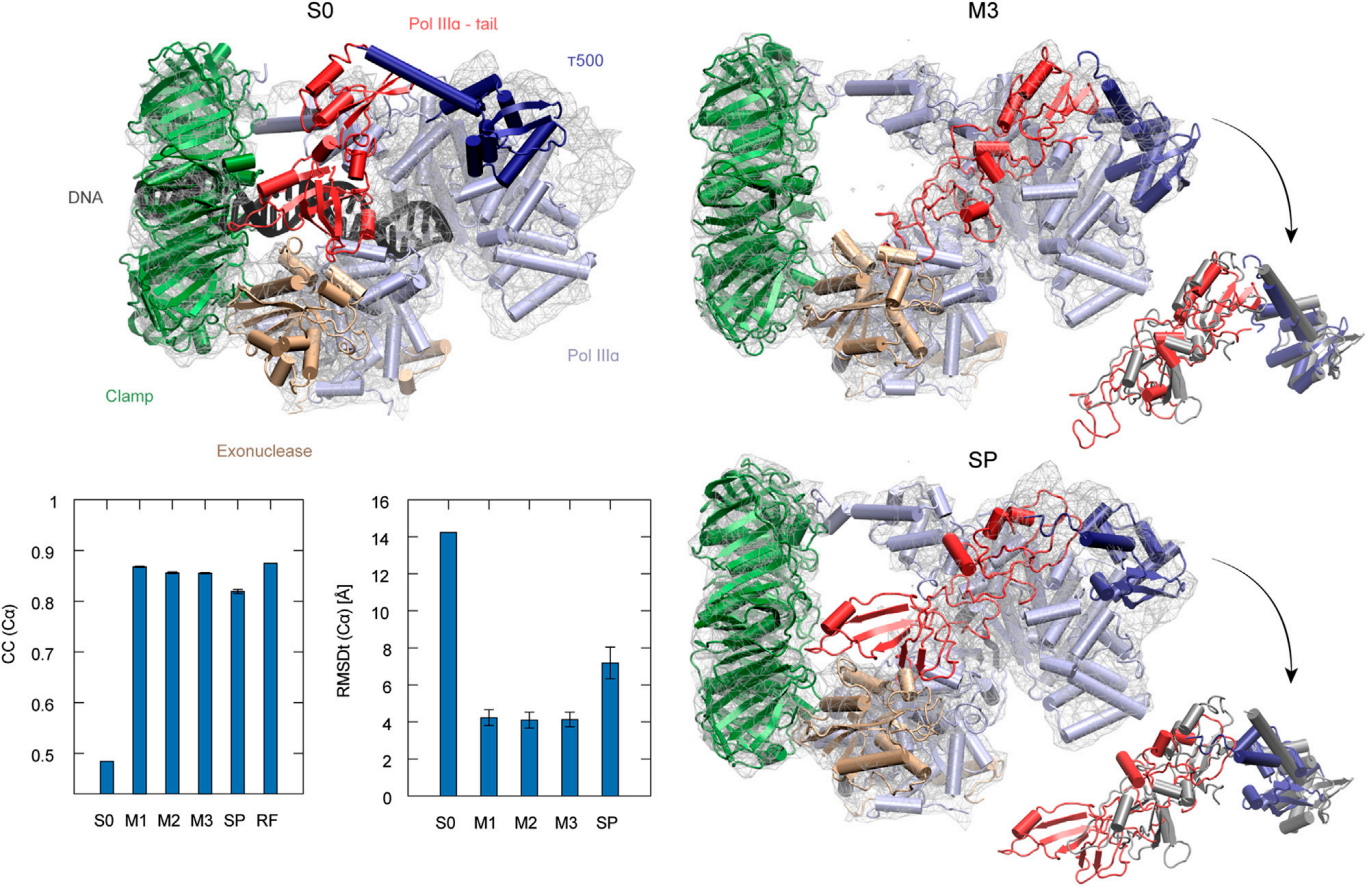
DNA polymerase fitting. S0: Initial structure with the target density map. Position of DNA is shown in black (not included in simulations). M3 and SP are the highest CC models obtained with the multi-scale and simple protocols, respectively. τ500 and tail of PolIIIα are additionally superimposed with the reference structure (RF) in dark gray in the insets. The average CC and RMSDt for 5 best models in each run are shown in the graphs with standard deviation marked as error bars.

The second target using the experimental density map is the DNA polymerase III from *E. coli*. It serves as an enzyme for DNA replication. The complex consists of several factors of different functionality, such as the clamp for sliding along the DNA and exonuclease working as a proofreader. To build the DNA lagging strand, the polymerase needs to quickly and repeatedly release and reposition DNA. In order to understand this process, several structures of the complex have been solved, both in DNA-bound and DNA-free states (Fernandez-Leiro et al., 2015). Here, we performed flexible fitting to a density map of a DNA-free state starting from the DNA-bound state structure. DNA was removed from the original PDB structure before the simulations (Dark gray in S0 panel of Figure 5).

The best model obtained with the simple protocol reveals many inaccuracies with respect to the density map in the regions of the τ500 domain and neighboring tail of PolIIIα, when compared with the modeled reference structure (SP panel in Figure 5). Particularly, the tail of PolIIIα that undergoes a rearrangement upon DNA binding is not in a correct position, significantly stretching out of the contours of the density map. Applying the multi-scale protocol allowed avoiding such problems. The secondary structure of those domains is retained with their simultaneous shift toward the correct position within the target density map (M3 panel in Figure 5). Both CC and RMSDt of the Cα atoms of the best final models are significantly improved by using the multi-scale protocol, from 0.82 to 0.85 and from 7.2 to 4.1, respectively (bottom left panel in Figure 5). Similarly, as in the case of the CorA, the CG simulations with REUSfit largely contribute to the correct fitting.

### Overall Performance of Multi-Scale Protocol

In Tables 2, 3 and Supplementary Table S1, we summarize the CC, RMSD, and other parameters of the best models obtained from the simple and multi-scale protocols for all test sets. In the cases of Adenylate Kinase, Maltodextrin Binding Protein, Ribose Binding Protein, and CO Dehydrogenase, which include a simple rotation or shift of the component domains, the two protocols are almost comparable. However, in the case of Ca^2+^-ATPase, Na^+^ K^+^-ATPase, Diphtheria Toxin, Magnesium Transporter, and DNA polymerase, which includes complicated conformational changes, multi-scale protocol showed better scores than the simple protocol. In the simple protocol, RMSDt of all the heavy atoms is high, reaching even 9.2 Å for Ca^2+^-ATPase and 10.3 Å for Diphtheria Toxin. In some cases, we also observe disruptions of the secondary structures in the rotated/shifted domain, resulting in higher secondary structure scores (>30%), and thus, poor MolProbity score and high Ramachandran and CaBLAM outliers. In the multi-scale protocol, RMSDt came down below 2.5 Å in all systems with simulated density maps and to 2.9 and 4.5 Å in the experimental density maps systems. The secondary structure scores are mostly less than 30%, and the main chain geometry is consistently at least 0.5% better than for the SP best models. We suggest that multi-scale protocol is useful for large proteins or their complexes in which a complex conformational transition, such as a rotation or domain rearrangement, occurs between two states.

**TABLE 3:** Quality of the best models obtained using the simple and multi-scale protocol for the experimental density maps.

System	Str.	c.c.	RMSDt (Cα) [Å]	RMSDt (AA) [Å]	MolProbity score	Ramachandran outlier [%]	Rotamer outlier [%]	CABLAM outlier [%]	Sec. struct. index [%]
Magnesium transporter CorA	MS3	0.77 (0.77 ± 0.00)	1.7 (1.8 ± 0.1)	2.9 (3.0 ± 0.0)	1.70 (1.70 ± 0.02)	2.8 (3.0 ± 0.6)	7.3 (7.3 ± 0.4)	5.3 (4.5 ± 0.5)	18 (19 ± 1)
	SP	0.74 (0.74 ± 0.00)	4.4 (4.5 ± 0.1)	5.1 (5.1 ± 0.1)	1.75 (1.76 ± 0.01)	2.9 (3.3 ± 0.5)	7.2 (7.7 ± 0.3)	4.8 (5.9 ± 0.7)	22 (22 ± 1)
DNA polymerase	MS3	0.85 (0.85 ± 0.00)	4.1 (4.1 ± 0.4)	4.5 (4.5 ± 0.3)	1.84 (1.80 ± 0.03)	3.6 (3.6 ± 0.4)	9.3 (8.2 ± 0.7)	6.7 (6.3 ± 0.4)	21 (21 ± 1)
	SP	0.82 (0.82 ± 0.00)	6.3 (7.2 ± 0.8)	6.6 (7.5 ± 0.8)	1.84 (1.81 ± 0.02)	4.4 (3.9 ± 0.4)	8.5 (8.1 ± 0.4)	6.1 (6.5 ± 0.3)	23 (24 ± 1)

## Discussion

To date, several methods relying on MD simulations have been developed in order to flexibly fit protein structures to the target density maps. Here, we use the CC-based approach (Tama et al., 2004). This can be combined with structure-based force fields, as in MDfit (Whitford et al., 2011), which enables us to prevent the structure from breaking secondary or tertiary structure contacts during the fitting due to native contact energy term. However, new stable contacts cannot be created during the fitting, even if there is a contact in the target structure. MDFF uses the physics-based force field such as CHARMM, which allows for creating new contacts, but requires secondary structure restraint (Villa and Lasker, 2014) (Trabuco et al., 2008). In our approach, we aim to design a fitting method that does not only keep secondary and tertiary structure relations, but also allows for the refinement of side chain positions, including breaking or creating new contacts. Therefore, we use a structure-based model for the first stage of the protocol, and CHARMM force field with the CC-based biasing potential for the third stage, joined together by targeted MD. One advantage of our method is that in the second and third stages we do not introduce any secondary structure restraints, as in CC-based methods, but we take advantage of those restraints at an earlier step by using the structure-based model. Although the protocol requires the correct secondary structure in the initial model, it allows the changes of the secondary structure in the target structures via the non-restraint MD simulations.

Another advantage of our protocol is that we are searching a large conformational space in the first stage with REUSfit (Miyashita et al., 2017), where the computational cost is reduced with the CG model. REUSfit increases the reliability of the fitting, since exchanging the force constants between replicas can promote a feedback loop to output the structures with the highest CC in different fitting trials. Extending the normal MD simulation or using other methods increasing the sampling such as accelerated MD would also lead to a wide conformational search (Hamelberg et al., 2004). However, it may not choose and promote the same fitted pose in many runs when dealing with a complex conformational transition.

For systems with large and complex conformational changes, it is difficult to obtain reliable results just using a simple method, as the results may not be converged (Ahmed et al., 2012). Frequently, wrong fitting poses are obtained, similarly as in the case of Diphtheria Toxin presented here. Thus, for such cases, our multi-scale approach combining CG and AA model is powerful to reach a final model with reliable backbone positions by REUSfit and also containing hydrogen atoms. The consideration of the hydrogen atoms and hydrogen bonds is of importance for proteins and it allows for refinement of the side chains. Introducing a protocol consisting of three steps may be seen as a disadvantage of our method due to the relative complexity of setting up the files for both CG and AA simulations and running 3 different types of simulations consecutively in comparison with a single flexible fitting run. All necessary functionalities are implemented within the GENESIS suite of programs (Jung et al., 2015) (Kobayashi et al., 2017) (Mori et al., 2019).

The protocol uses targeted MD to convey the information about the correct Cα atoms positions after structure-based model fitting to the AA simulations. The most straightforward way one could imagine for going from Cα only model to AA model would be to rebuild the protein backbone and side chains basing on conventional geometrical principles. Various tools were examined in step 2, including for example PULCHRA (Rotkiewicz and Skolnick, 2008) and SCWRL4 (Krivov et al., 2009). In our experiences, none of them was free of severe errors in rebuilt structures, especially in the case of the slightly distorted main chain after flexible fitting simulation. Common problems included strange dihedral angles of the backbone, overlapping side chains and ring penetration (i.e., insertion of a covalent bond into an aromatic ring). We consider that targeted MD is generally applicable to back mapping in multi-scale simulations that combine CGMD and AAMD when at least one reliable experimental structure is available. Another method that could be taken into consideration for retrieving atomic detail from CG model is a recently developed back mapping method using Bayesian inference and restrained MD (Peng et al., 2019).

In this study, we tried to obtain the one best model from one experimental density map. Understanding the experimental data as an ensemble average of the multiple conformations is also important, since many conformations can reproduce the same experimental data (degeneracy problem) (Rieping et al., 2005) (Cossio and Hummer, 2013) (Olsson et al., 2017). In the cryo-EM, multiple states of proteins can be classified with the 3D reconstruction protocol for a large amount of 2D images (Bai et al., 2015). However, the obtained 3D density map is still an ensemble average of molecules with sufficient conformational fluctuations in solution. In addition, noise or random errors might be contained in the map to some extent, even if high-resolution density maps are obtained. To address these issues in the structure modeling, various inferential algorithms have been proposed (Bonomi et al., 2017). Particularly, metainference calculates the ensemble average using multiple-replica simulations, and also considers effects of the noise and errors in the energy function based on the Bayesian inference (Bonomi et al., 2016), which was recently applied to the cryo-EM structure modeling (Bonomi et al., 2018). Introduction of the ensemble average, noise, or errors will be one of the future extensions of our protocol.

## Conclusion

We have designed a multi-scale protocol by integrating the CGMD with the AAMD in a single flexible fitting workflow. Performed simulations for several systems containing intricate domain rotations, domain shifts and transitions between open and closed states show that we can improve the accuracy and reliability of the flexible fitting procedure in comparison with a single, long AA flexible fitting simulation. The protocol performs well both for simulated and experimental density maps from cryo-EM and it is recommended to use with large proteins or their complexes.

## Data Availability

The original contributions presented in the study are included in the article/Supplementary Material, further inquiries can be directed to the corresponding author. The simulation protocol will be available as a tutorial “Multi-scale Cryo-EM flexible fitting” at the GENESIS webpage (https://www.r-ccs.riken.jp/labs/cbrt/).
