# Supplementary material for: Multi-Scale Flexible Fitting of Proteins to Cryo-EM Density Maps at Medium Resolution
Source: Front Mol Biosci. 2021 Mar 19;8:631854. doi: 10.3389/fmolb.2021.631854 (PMC8025875; doi:10.3389/fmolb.2021.631854)
Supplement: Supplementary file 3 [file table1.pdf]

Supplementary Material

Multi-Scale Flexible Fitting of Proteins to Cryo-EM Density Maps at Medium Resolution

Marta Kulik[1#], Takaharu Mori[1], Yuji Sugita[1,2,3*]

[1] Theoretical Molecular Science Laboratory, RIKEN Cluster for Pioneering Research, 2-1 Hirosawa, Wako-shi, Saitama 351-0198, Japan

[2] RIKEN Center for Computational Science, 7-1-26 Minatojima-minamimachi, Chuo-ku, Kobe, Hyogo 650-0047, Japan

[3] RIKEN Center for Biosystems Dynamics Research, 7-1-26 Minatojima-minamimachi, Chuo-ku, Kobe, Hyogo 650-0047, Japan

Correspondence:
Yuji Sugita

sugita@riken.jp

Present address: Biological and Chemical Research Centre, Department of Chemistry, University of Warsaw, ul. Żwirki i Wigury 101, 02-089 Warsaw, Poland

Movie S1.

Correct fitting of domain A is obtained in the multi-scale protocol. Flexible fitting simulation for Ca^{2+}-ATPase from E2 to E1·2Ca^{2+} state. Coarse-grained structure, all-atom structure and density map are shown as red ribbon, colorful cartoon and grey wireframe, respectively. For coloring of domains, see Figure 2. In the last snapshots, the fitted model is superimposed with the target structure in dark grey.

Movie S2.

Domain A cannot be fitted correctly using the simple protocol. Flexible fitting simulation for the same system as in Movie S1. Domains in all-atom structure are colored as in Figure 2. In the last snapshots, the fitted model is superimposed with the target structure in dark grey.

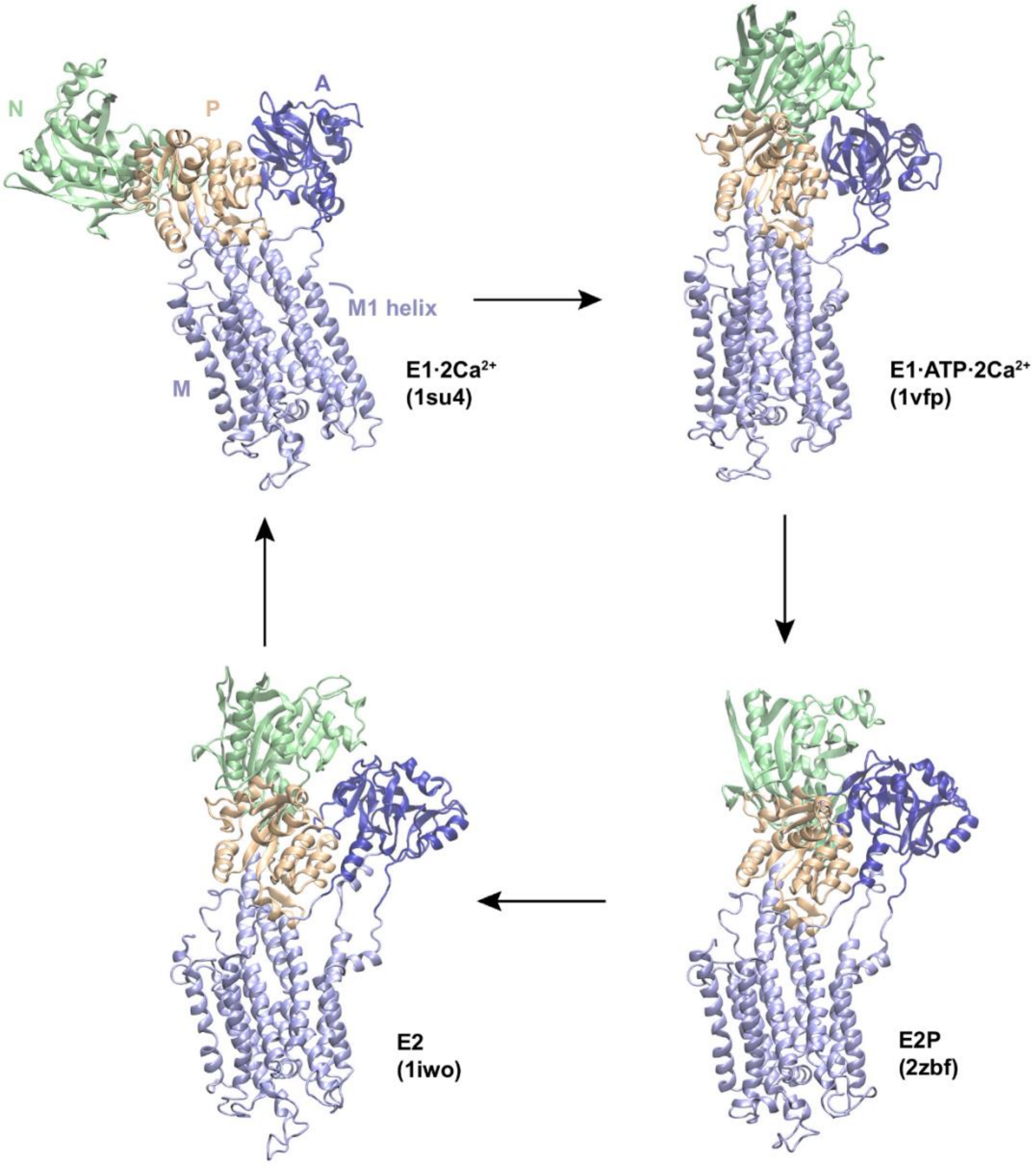

Figure S1. Physiological states of Ca^{2+}-ATPase used in the flexible fitting simulations. The orientation of each structure mimics the orientation in the membrane (Norimatsu et al., 2017).

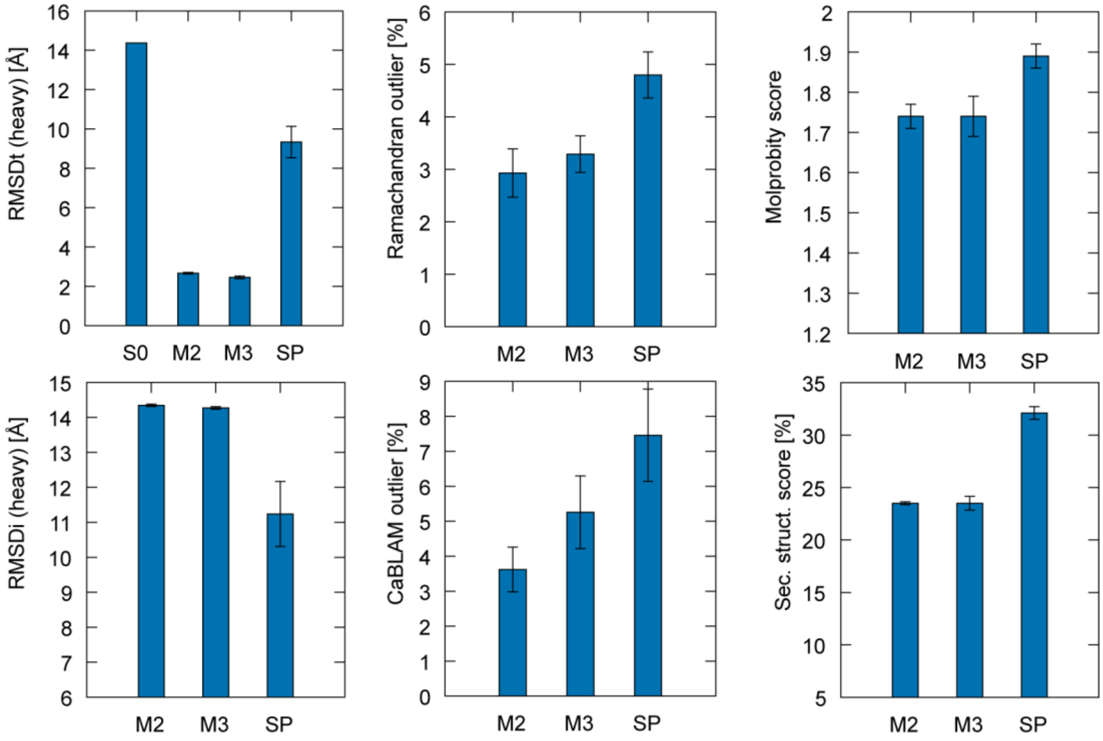

Figure S2. The average value for the 5 best models in targeted MD and flexible fitting simulations of Ca^{2+}-ATPase from E2 to $E1{\cdot}2Ca^{2+}$ state. The standard deviation is shown as error bar.

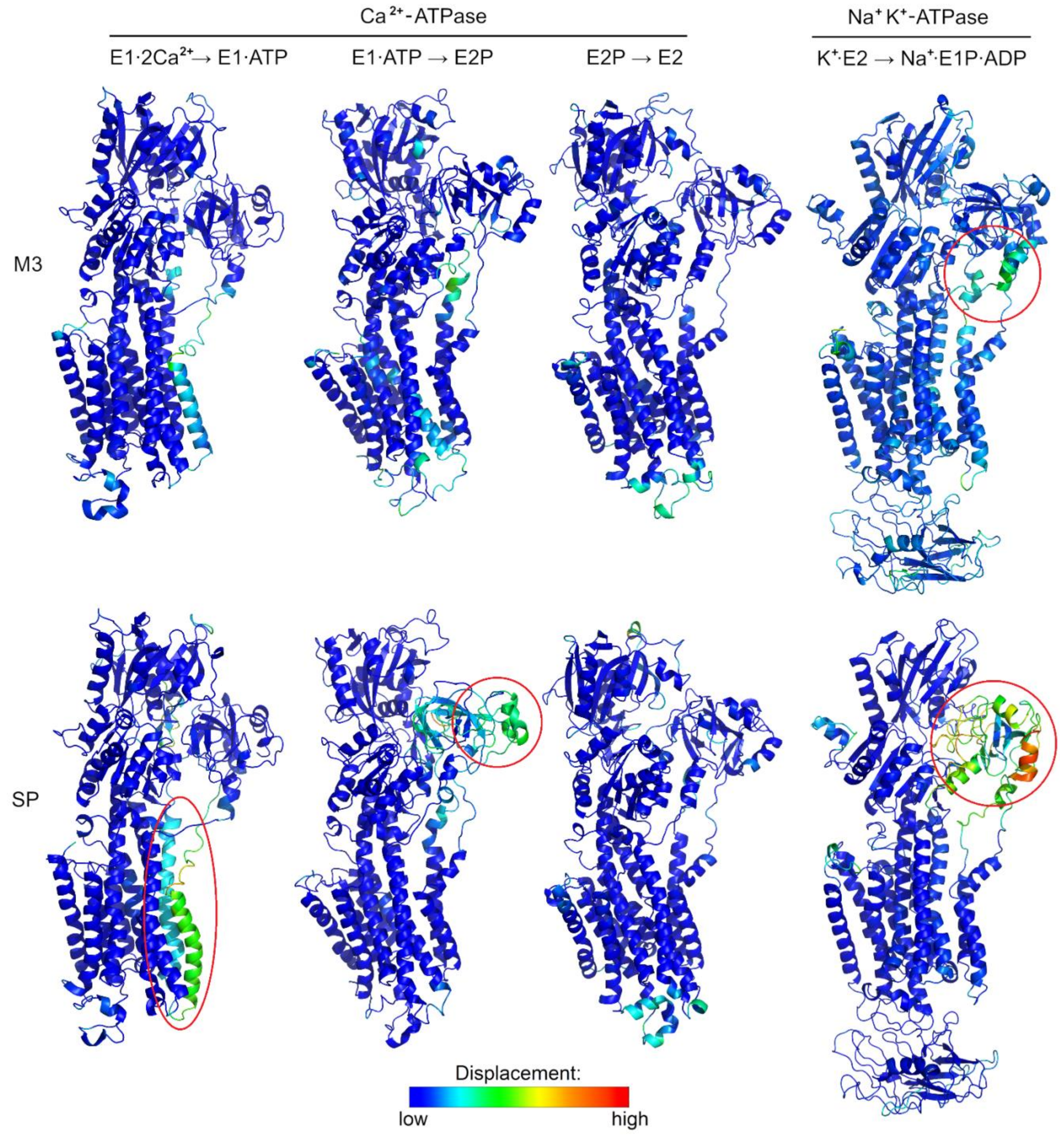

Figure S3. The highest CC models colored according to the distance between the Cα atoms of the target and the shown structure. The parts with lowest to highest deviation are shown in blue to red, respectively.

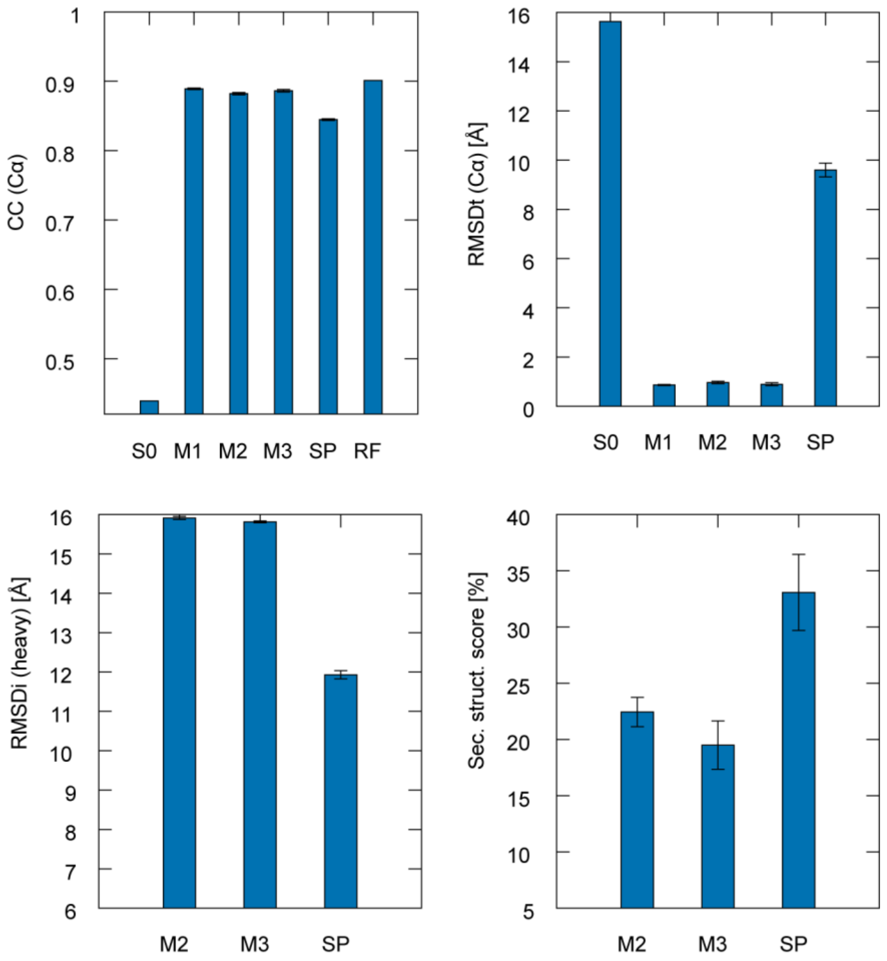

Figure S4. Simulations of Diphtheria Toxin. The average value for the 5 best models in REMD simulations and 5 best models in targeted MD and flexible fitting. The standard deviation is shown as error bar.

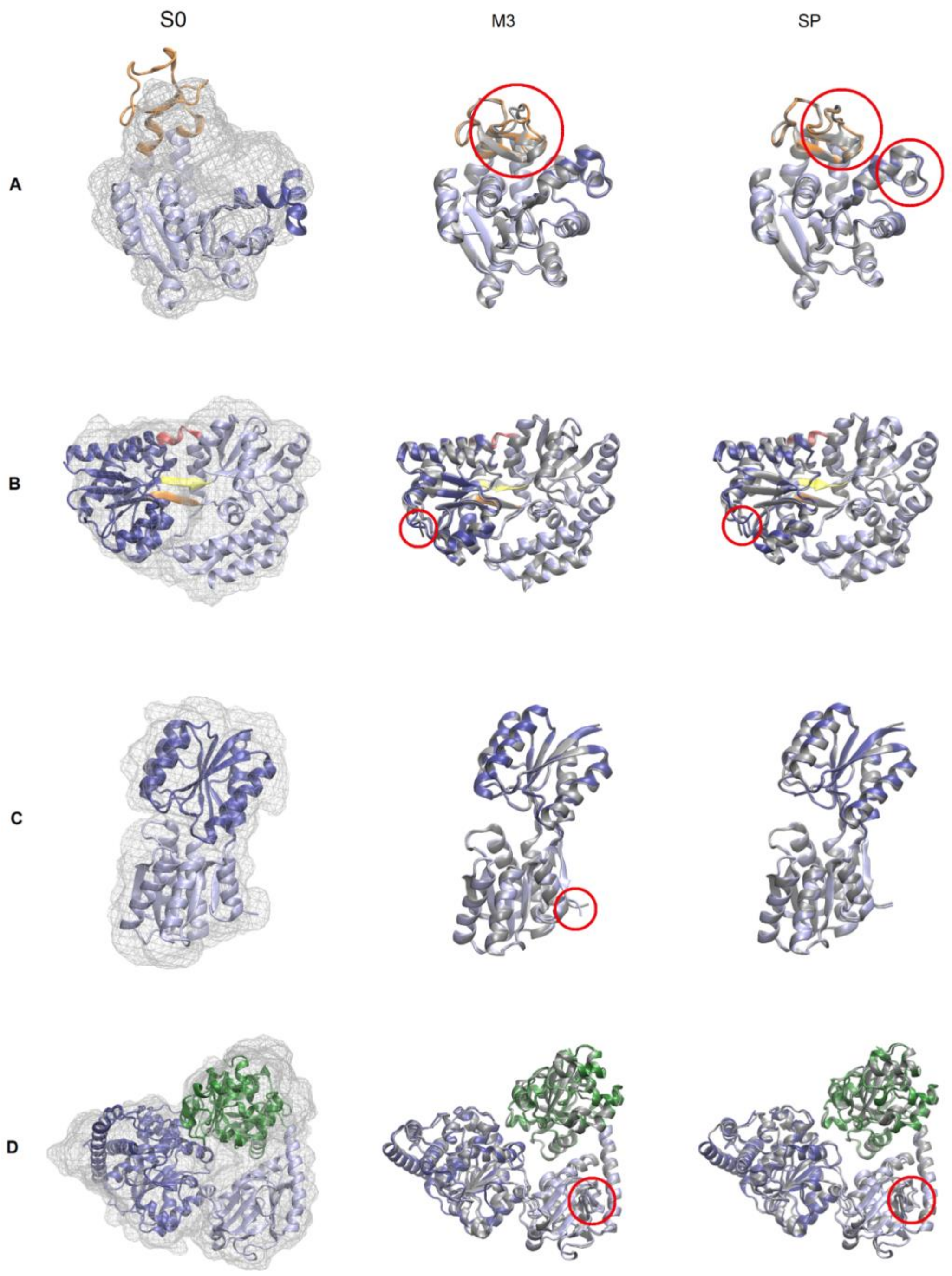

Figure S5. Systems for which multi-scale and simple protocols give comparable results. Initial structure S0 with the target density map and the highest CC models obtained using the multi-scale (M3) and simple (SP) protocols are shown for (A) Adenylate Kinase, (B) Maltodextrin Binding Protein, (C) Ribose Binding Protein and (D) CO Dehydrogenase. Larger deviations from the target structure (in grey) are circled.

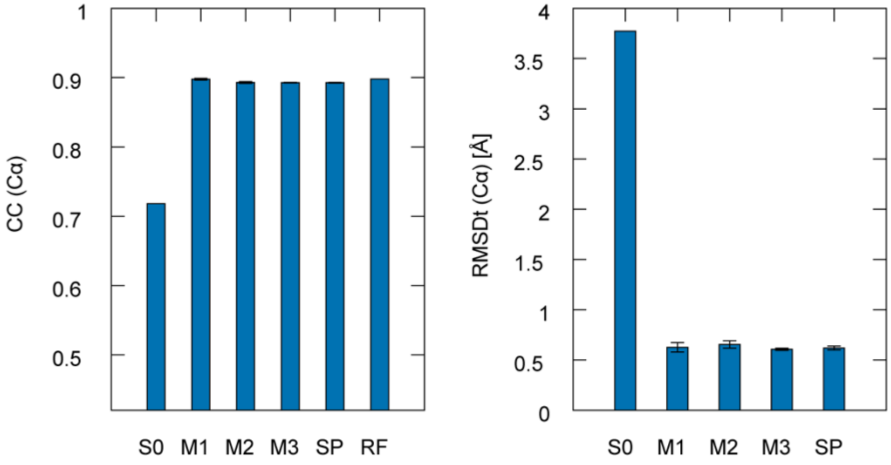

Figure S6. Maltodextrin Binding Protein simulations. The average value for the 5 best models in REMD simulations and 5 best models in targeted MD and flexible fitting simulations. The standard deviation is shown as error bar.

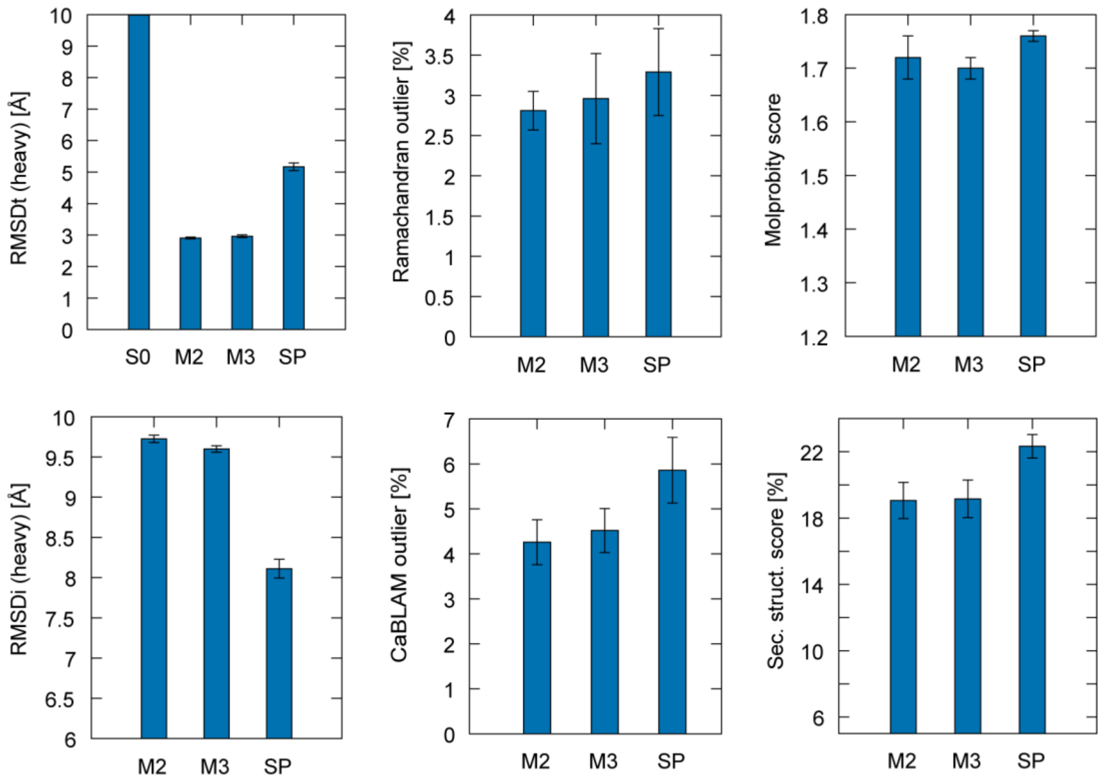

Figure S7. CorA simulations. The average value for the 20 best models in REMD simulations and 5 best models in targeted MD and flexible fitting simulations. The standard deviation is shown as error bar.

Table S1. Quality of the best models obtained using the simple and multi-scale protocol for the simulated density maps for Na^+ K^+ ATPase, Adenylate Kinase, Ribose Binding Protein, Maltodextrin Binding Protein and CO Dehydrogenase.

**System**	Str.	c.c.	RMSDt (Cα) [Å]	RMSDt (AA) [Å]	MolProbity score	Ramachandran outlier [%]	Rotamer outlier [%]	CABLAM outlier [%]	Sec. struct. index [%]
Na^+ K^+ ATPase									
K^+·E2→ Na^+·E1P·ADP	MS3	0.95 (0.95±0.00)	1.5 (1.6±0.1)	2.2 (2.3±0.1)	1.84 (1.81±0.04)	2.9 (3.0±0.6)	9.9 (8.5±0.7)	5.9 (5.8±0.6)	24 (24±1)
	SP	0.92 (0.92±0.00)	6.1 (5.8±0.2)	6.4 (6.1±0.2)	1.84 (1.86±0.03)	4.8 (4.0±0.6)	8.6 (9.2±0.6)	8.1 (7.6±0.6)	26 (26±1)
Na^+·E1P·ADP → K^+·E2	MS3	0.95 (0.95±0.00)	1.6 (1.7±0.0)	2.4 (2.4±0.1)	1.70 (1.77±0.03)	4.2 (3.4±0.3)	6.6 (8.2±1.1)	4.6 (5.0±0.6)	29 (30±0)
	SP	0.92 (0.91±0.01)	4.7 (5.5±1.4)	5.0 (5.8±1.4)	1.75 (1.82±0.05)	3.7 (3.7±0.5)	6.8 (8.4±0.9)	6.9 (6.1±1.0)	34 (34±1)
Adenylate Kinase	MS3	0.99 (0.99±0.00)	0.8 (0.7±0.1)	1.3 (1.4±0.1)	1.71 (1.68±0.04)	1.4 (1.7±0.2)	9.3 (6.6±3.5)	3.8 (2.3±1.0)	14 (14±2)
	SP	0.99 (0.99±0.00)	0.5 (0.6±0.1)	1.0 (1.2±0.1)	1.46 (1.60±0.10)	0.9 (1.2±0.8)	6.2 (7.5±1.1)	1.4 (2.6±1.3)	10 (15±3)
Ribose Binding Protein	MS3	0.99 (0.99±0.00)	0.5 (0.5±0.0)	1.0 (1.0±0.0)	1.39 (1.34±0.11)	1.2 (1.5±0.3)	5.2 (5.6±1.0)	1.5 (1.8±0.6)	7 (9±1)
	SP	0.99 (0.99±0.00)	0.5 (0.5±0.0)	0.9 (0.8±0.0)	1.39 (1.23±0.08)	1.9 (1.2±0.5)	6.2 (3.8±1.2)	1.9 (1.6±0.3)	8 (8±1)

Maltodextrin Binding Protein	MS3	0.99 (0.98±0.00)	0.6 (0.6±0.0)	1.2 (1.2±0.1)	1.61 (1.64±0.05)	1.4 (1.4±0.6)	6.6 (7.7±1.1)	1.4 (1.5±1.0)	10 (12±2)
	SP	0.99 (0.99±0.00)	0.7 (0.6±0.0)	1.1 (1.2±0.0)	1.65 (1.60±0.08)	0.5 (1.2±0.4)	10.6 (8.0±1.4)	1.6 (1.9±0.6)	10 (9±3)
CO Dehydrogenase	MS3	0.98 (0.98±0.00)	0.8 (0.8±0.0)	1.4 (1.4±0.0)	1.67 (1.69±0.04)	1.5 (2.3±0.6)	6.9 (7.1±0.7)	4.0 (3.6±0.2)	13 (13±1)
	SP	0.98 (0.98±0.00)	0.7 (0.8±0.0)	1.2 (1.3±0.1)	1.66 (1.65±0.03)	1.2 (1.8±0.6)	6.8 (6.8±0.6)	3.9 (3.7±0.4)	12 (12±1)
